# Late resolution of cow’s milk and egg allergy: experience at a third level centre

**DOI:** 10.1186/2045-7022-1-S1-P31

**Published:** 2011-08-12

**Authors:** Francesca Barbon, Francesca Lazzarotto, Alice Toniolo, Antonella Muraro

**Affiliations:** 1 Food Allergy Centre, Padua University Hospital, Dept. of Pediatrics, Padova, Italy

## Background

Cow's milk allergy affects about 2-3 % of general population; the prevalence of egg allergy is 1-2% among childhood. According to the literature the development of tolerance for cow’s milk and for egg is reached in early childhood, in the majority of case. In addiction it is reported that over six years of age there is a significant decrease in the resolution rate both for cow’s milk and egg allergy.

## Objective

The aim of this study is to define the rate of allergy resolution over time in a selected population of cow's milk and egg allergy patients.

## Methods

We conducted a retrospective study on 110 patients, 42 with cow’s milk and 68 with egg allergy, which attend the Food Allergy Centre, Padua, Veneto. All the patients studied were between 5 and 15 years and they had not yet developed tolerance to egg or milk. The lack of tolerance was confirmed by an oral food challenge (OFC). A descriptive analysis was conducted on the collected data.

## Result

The development of tolerance was reached in 66,7% (28) patients with cow’s milk allergy, in particular 57.1% (16) from 6 to 7 years old, 17.8% (5) from 8 to 9 years old, 7.2% (2) from 10 to 11 years old, 14.3% (4) from 12 to 13 and 3.6% (1) from 14 to 15. The total percentage of patients who achieved tolerance to egg was 67.6%. The distribution among the different groups of age is as follow: 58.7% (27) from 6 to 7 years old, 13.1% (6) from 8 to 9 years old, 15.2% (7) from 10 to 11 years old, 6.5% (3) from 12 to 13 and 6.5% (3) from 14 to 15.

## Conclusion

Our data show that achieving of tolerance is still possible for both cow's milk and egg at ages older than six years. The rates of resolution for both cow's milk and egg allergy between different rising age groups are similar. In spite of several limitations for a non homogeneous and non proportional distribution of patients between the different ages, the results underline the importance of monitoring the older children with OFC during years at, at least, yearly intervals as some of these children can still outgrow their food allergy.

